# Supplementary material for: Bridging between Mouse and Human Enhancer-Promoter Long-Range Interactions in Neural Stem Cells, to Understand Enhancer Function in Neurodevelopmental Disease
Source: Int J Mol Sci. 2022 Jul 19;23(14):7964. doi: 10.3390/ijms23147964 (PMC9322198; doi:10.3390/ijms23147964)
Supplement: Supplementary file 1 [file S1-S5.pdf › S1-S5.pdf]

Figure S1

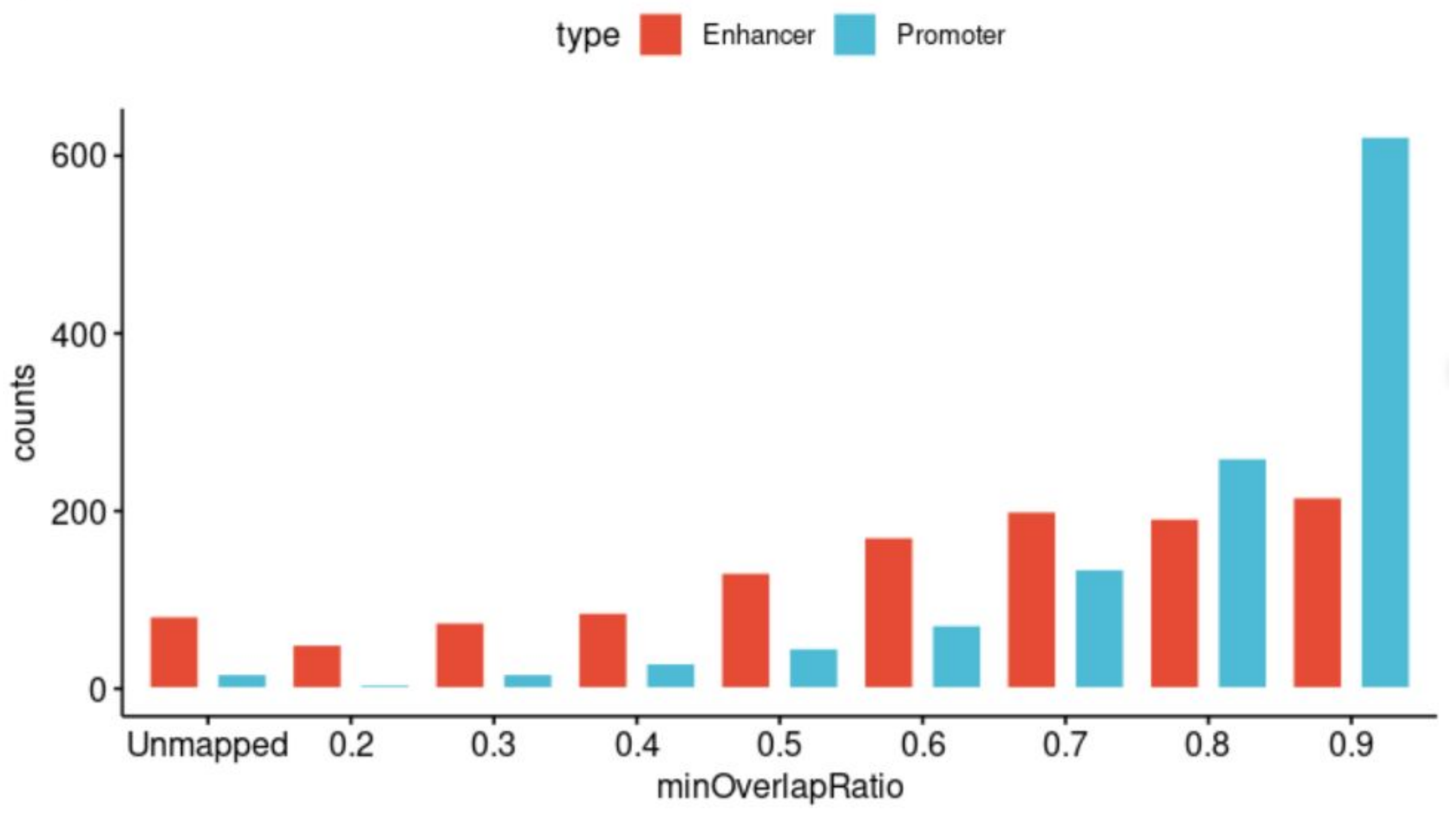

Figure S1. Number of mouse enhancer/promoter anchors of wTR1 experiment which were mapped to human genome by liftOver, foreach percentage of overlap (0.2 to 0.9). Unmapped anchors were also shown. Promoter anchors generally mapped at higher overlap level than enhancer

Figure S2

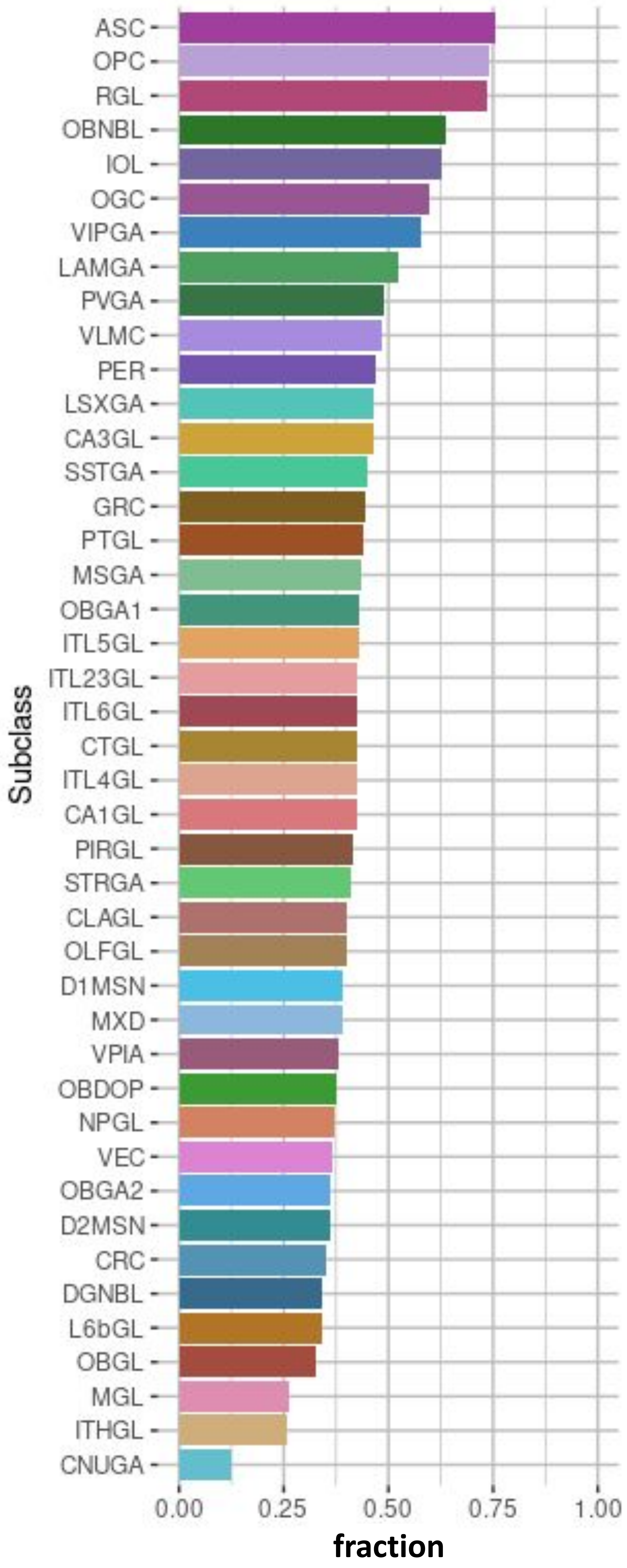

Figure S2
Sorted histograms showing the relative proportion of cCREs (Li et al. 2021) overlapping our mouse enhancers connected to promoters via LRI, for each of the 43 cell type subclasses. For abbreviations of cell subclasses names, see (Li et al. 2021).

Figure S3

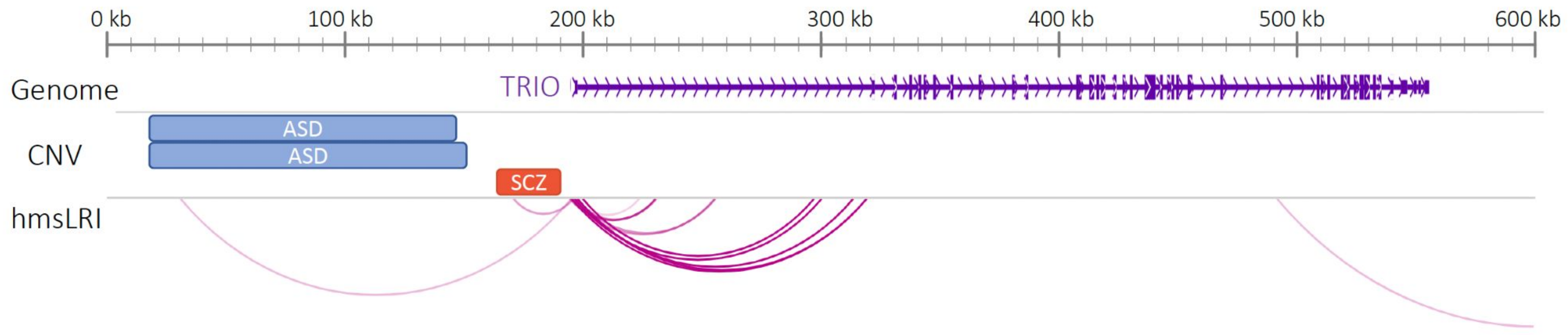

Figure S3
CNV associated to autism spectrum disorder (ASD; copy number gain, blue rectangle) or schizophrenia (SCZ; copy number loss, red rectangle) overlap with human putative enhancers connected to the TRIO gene promoter by the indicated hmsLRI.

Figure S4

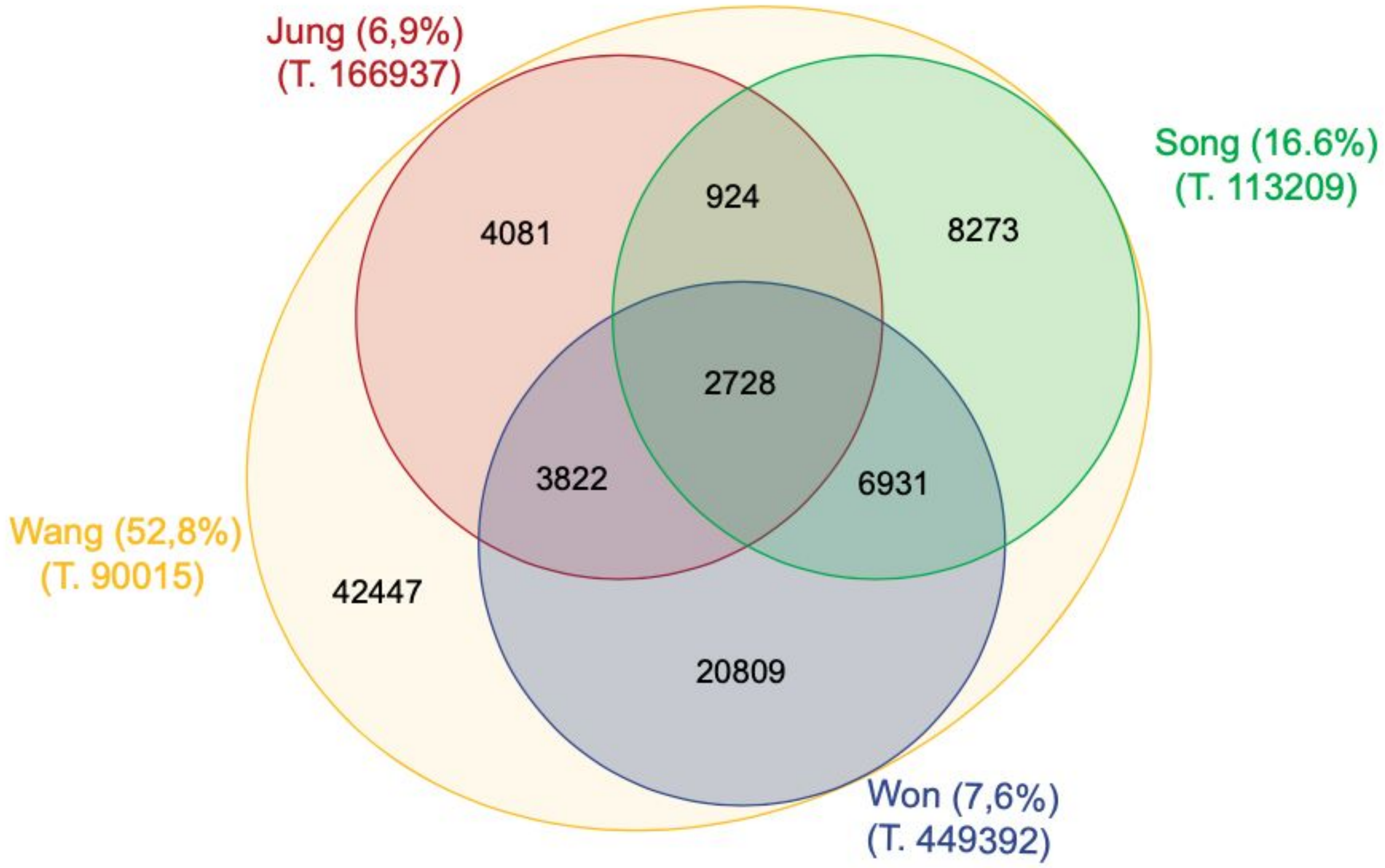

Figure S4
The Venn diagram shows how many human HiC-derived enhancer-promoter interactions from PsychENCODE Consortium (Wang et al 2018) were also identified by three independent studies (Won et al 2016, Jung et al 2019 and Song et al 2020). For each study, the total number of interactions and the percentage of those in common with the rest of the studies are shown in parentheses.

Figure S5

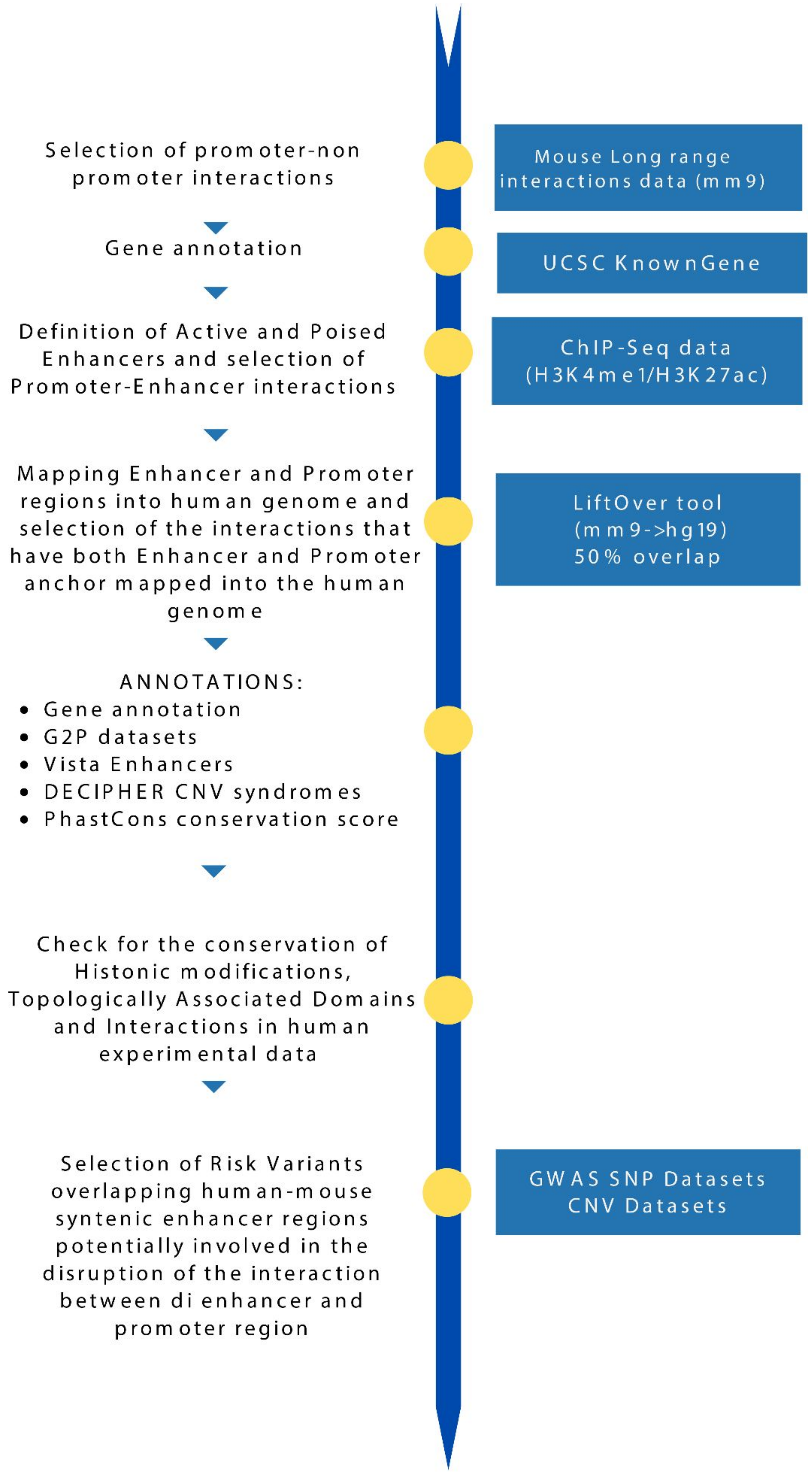

Figure S5
schematic workflow with details on analysis strategy, tools and database resources.
